# The Effects of Beta-Hydroxy-Beta-Methylbutyrate-Free Acid Supplementation and Resistance Training on Oxidative Stress Markers: A Randomized, Double-Blind, Placebo-Controlled Study

**DOI:** 10.3390/antiox7060076

**Published:** 2018-06-11

**Authors:** Hamid Arazi, Abbas Asadi, Katsuhiko Suzuki

**Affiliations:** 1 Department of Exercise Physiology, Faculty of Sports Sciences, University of Guilan, Rasht 1438, Iran; Abbas_asadi1175@yahoo.com; 2 Faculty of Sport Sciences, Waseda University, Tokorozawa 359-1192, Japan; katsu.suzu@waseda.jp

**Keywords:** leucine, strength training, free radicals, oxidative stress

## Abstract

The aim of this study was to investigate the effects of 6-week beta-hydroxy-beta methylbutyrate-free acid (HMB-FA) supplementation on oxidative stress and biochemical variables in responses to resistance training. Sixteen healthy young males participated in this study and were randomly assigned to a HMB-FA supplementation group (*n* = 8) or a placebo supplementation group (*n* = 8). The resistance training program was applied for 6 weeks with two sessions per week. Blood samples were collected before and after training, and 8-hydroxy-2-deoxyguanosine (8-OHdG), malondialdehyde (MDA), protein carbonyl (PC), and biochemical variables, such as alanine transaminase (ALT), aspartate aminotransferase (AST), alkaline phosphatase (ALP), and the numbers of total white blood cells (WBC), neutrophils, lymphocytes, and monocytes were analyzed. Following intervention, both the HMB-FA and placebo supplementation groups showed significant decreases in MDA (effect size [ES]; −0.39, −0.33) and PC (ES; −1.37, −1.41), respectively. However, 8-OHdG did not change after 6 weeks of training in any of the groups. In addition, both groups showed similar training effects on biochemical variables after 6 weeks of intervention. It was concluded that HMB-FA supplementation during resistance training did not add further adaptive changes related to oxidative stress markers.

## 1. Introduction

It has been well documented that both aerobic and anaerobic exercise result in neutrophil translocation to the active skeletal muscle, subsequently resulting in oxidative stress [1]. Although an acute bout of exercise could result in activation of several systems of free radical generation (i.e., burst of neutrophils) [2], regular exercise training promotes antioxidant defenses and exerts anti-inflammatory effects (i.e., resting redox status) on the blood and skeletal muscle [3].

Most of the experimental studies that have investigated the effects of exercise training on oxidative stress have used endurance training, and only a few studies have investigated the effects of resistance training on oxidative stress adaptations, showing that long term resistance training has protective effects, including the enhancement of defense systems (i.e., superoxide dismutase) [4,5,6,7].

It is well known that antioxidant supplementation can inhibit the induction of endogenous antioxidant capacity in response to exercise training [6,7]. On the other hand, antioxidant supplementation among athletes is common; however, there is little information regarding whether amino acid supplementation protects against the negative health consequences of reactive oxygen species (ROS), such as oxidative DNA damage, lipid peroxidation, and protein modification caused by resistance training in men [8]. 

Leucine, a branched-chain amino acid, is a potent stimulus for translation initiation and protein synthesis [9]. Leucine is also believed to have immunomodulatory effects [10] which may be mediated by its metabolites [11]. Beta-hydroxy-beta-methylbutyrate (HMB) is an intermediate of leucine metabolism and has been shown to increase protein synthesis while simultaneously decreasing muscle proteolysis [11]. It has been well documented that HMB supplementation decreases muscle damage, cytokine and inflammatory responses to exercise and chronic HMB supplementation has positive effects on muscle mass, body composition, and strength [11,12]. However, other studies have reported conflicting results and the results are controversial [13,14,15]. For example, different authors have demonstrated no change [16] or an increase in immune function in humans and an attenuation of catabolism and muscle damage via HMB supplementation [11,12,17,18]. Regarding the effects of HMB supplementation on immune cells, no studies have directly examined the effects of HMB supplementation on oxidative stress responses to resistance training. 

To the best of our knowledge, the majority of the studies have used a commercially available calcium salt form of HMB (HMB-Ca) for supplementation; however, a new free acid form of HMB (HMB-FA) has been found to yield higher plasma concentrations over a shorter period of time compared to the calcium salt form (36 vs. 131 min) [17]. The greater bioavailability of HMB-FA may provide greater benefits regarding its efficacy as a nutrient supplement used to enhance training adaptations and can promote the actions of defense system against oxidative stress and may play an important role in attenuating oxidative damage following exercise training. However, this is the first study to evaluate the effects of resistance training and HMB-FA supplementation on oxidative stress and biochemical variables in response to exercise training. Given the limited available information and few studies about the effects of HMB-FA supplementation in humans, especially the role of HMB-FA on oxidative stress following resistance training, the objective of this study was to evaluate the effects of chronic HMB-FA supplementation on oxidative stress markers and biochemical variables in response to 6 weeks of resistance training.

## 2. Materials and Methods

### 2.1. Experimental Design

This study had a placebo-controlled, double-blind, randomized design. All subjects were familiarized with the training program two weeks prior to the initiation of the study. Then, the subjects were recruited to the human performance laboratory to standardize training procedures. One week later the subjects were again recruited to laboratory for assessment of age, height and weight. For the prescription of the resistance training program, one repetition maximum test in each exercise was assessed on two non-consecutive days (i.e., leg press, bench press, knee extension and cable biceps curl on one day and knee flexion, lat pull-down, shoulder press, and triceps push down on another day, 48 h apart) at the same time of day, by the same investigators, using the Kraemer and Fry procedure [19]. Finally, forty-eight hours prior to the initiation of the training period, blood samples were taken in order to determine resting oxidative stress and biochemical variables. Forty-eight hours after the completion of the six-week training period, blood draws were again completed to determine the resting oxidative stress and biochemical variables.

### 2.2. Participants

**Figure 1:**
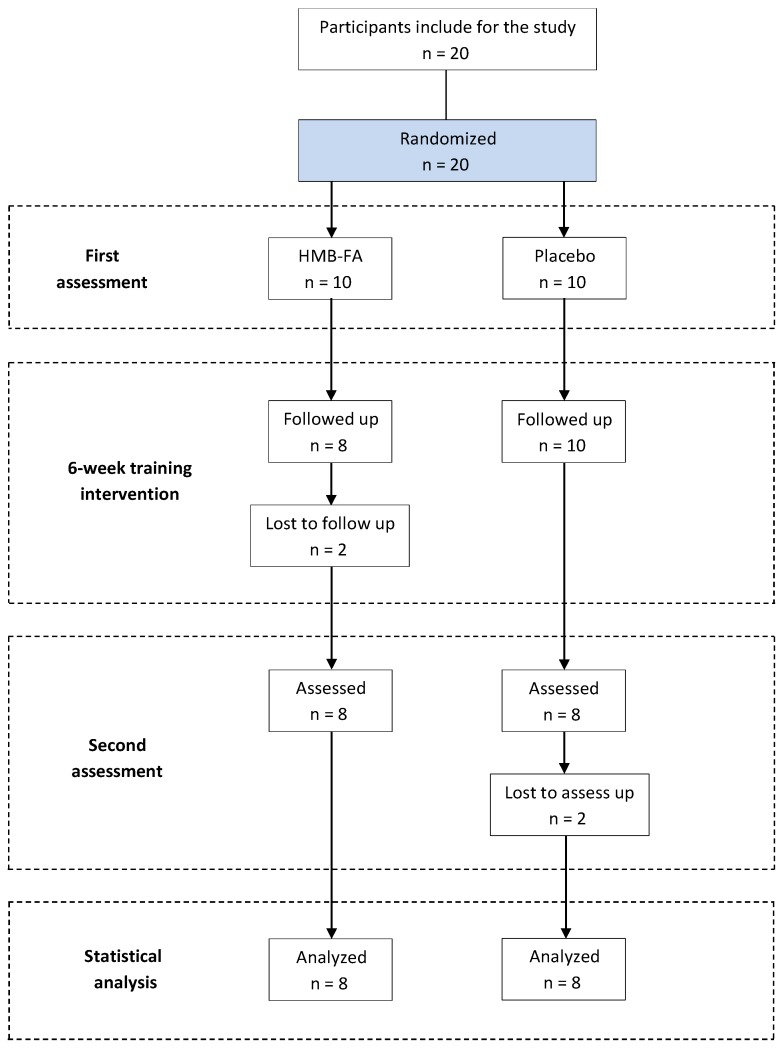
Study flow.

Initially, twenty healthy men volunteered to participate in this study. To be included in the final analyses, subjects were required to complete all of the training sessions and attend all assessment sessions. As a result of these requirements, four subjects were excluded from the study. Therefore, 16 men were included in the final analyses (Figure 1). For the final study subjects, the two study groups were as follows: beta-hydroxy-beta-methylbutyrate-free acid (HMB-FA) supplementation (*n* = 8, age = 21.5 ± 0.5 years, height = 181.7 ± 4.1 cm, body weight = 79.2 ± 13.0 kg, and BMI = 23.9 ± 3.2 kg/m^2^) and placebo supplementation (*n* = 8, age = 21.3 ± 0.9 years, height = 179.5 ± 4.5 cm, body weight = 78.9 ± 13.3 kg, and BMI = 24.4 ± 4 kg/m^2^). The subjects fulfilled the following inclusion criteria: (1) at least 1 year of resistance training experience determined by questionnaire and/or self-reported history by subjects; (2) had not used any drugs or supplements that could affect the results for 6 months before the initiation of study and throughout the study period; (3) 6 months with the absence of musculoskeletal injury or any orthopedic problems which could affect the efficiency of resistance training; and (4) no participation in other competitive sport activity aside from resistance training during the intervention period. Moreover, the subjects were familiar with the selected exercises in the resistance training program in their training routine and were asked to maintain their physical and diet habits throughout the experiment. The subjects were informed about the experimental procedures and possible risks and benefits associated with participation in the study, and they signed an informed consent before the start of the study. The study was conducted in accordance with the Declaration of Helsinki and was approved by the ethics committee of the University (DT-167908).

### 2.3. Measurements

Height was measured using a wall mounted stadiometer (Seca 222, Terre Haute, IN, USA) recorded to the nearest 0.5 cm; body weight was measured to the nearest 0.1 kg using a digital scale (Tanita, BC-418MA, Tokyo, Japan); and body mass index was calculated (kg/m^2^).

### 2.4. Blood Sampling

Blood samples were collected after 10 h of fasting, between 8 and 9 a.m., 48 h before, and 48 h after, the 6-week training intervention. A total of 10 mL of whole blood was drawn from the antecubital vein. The blood was collected into EDTA-containing tubes and centrifuged immediately at 1500× *g* for 10 min at 4 °C. The blood was allowed to clot at room temperature for 30 min, and the resulting serum was frozen at −80 °C for further analyses.

### 2.5. Biochemical Analyses

Serum 8-hydroxy-2-deoxyguanosine (8-OHdG), malondialdehyde (MDA), and protein carbonyl (PC) were analyzed using commercially available, enzyme-linked immunosorbent assays (ELISA) kits (ZellBio GmbH Veltlinerweg 29, 89075, Ulm, Germany). Serum alanine transaminase (ALT), aspartate aminotransferase (AST), and alkaline phosphatase (ALP) activities were measured using an automated analyzer (Model 747-400, Hitachi, Tokyo, Japan). Complete blood count analyses were conducted using an automated hematology analyzer (pocH-100i, Sysmex, Kobe, Japan), including the numbers of white blood cells (WBC), neutrophils, lymphocytes, and monocytes. All the assays were conducted in duplicate. The coefficient of variation for the measurements was less than 7%.

### 2.6. Diet Control

In order to examine whether dietary changes influenced oxidative stress variables, 3 consecutive days of diet recall were completed before beginning the training protocol [4,12]. At the end of the training period, subjects again recorded food intake for 3 consecutive days (before blood sampling). The mean daily calories, protein, carbohydrate, fat, vitamin C and vitamin E intakes during the 3 days assessed before and after the training intervention are presented in Table 1.

### 2.7. Supplementation

The HMB-FA supplement consisted of one gram of beta-hydroxy-beta-methylbutyrate in the free acid gel form (BetaTor, Body Attack, GmbH & Co., KG, Waldhofstrabe 19, 25474, Ellerbek, Germany). Each serving of placebo contained one gram of polydextrose. On training days, one serving of HMB-FA or placebo was consumed 30 min prior to the exercise session. Two servings were given with the breakfast and supper meals. On the non-training days, participants were instructed to consume one serving with each of three separate meals throughout the day. Subjects were required to return all used and unused packets at the end of each week.

### 2.8. Training Programe

The resistance training protocol consisted of a 6-week hypertrophic program involving 2 sessions per week. Each training session lasted 80 min, including 10 min of standard warm-up, 65 min of main training and 5 min of cool down. Subjects performed 3 sets of 8 to 12 repetitions with 75 to 85% of 1 RM (leg press, knee extension, knee flexion, lat pull-down, bench press, shoulder press, cable biceps curl and triceps push down) per session. The 2 and 3 min resting intervals were assigned between sets and exercises, respectively. All training sessions was monitored by Certified Conditioning Specialists in order to ensure that all exercises were performed correctly, with the appropriate amount of effort.

### 2.9. Statistical Analysis

Data are presented as means ± SDs. The distribution of each variable was examined using the Shapiro–Wilk test. The dependent variables were analyzed using 2 (group) × 2 (time) analysis of variance (ANOVA). Effect sizes (ES) were calculated to determine training effects. The magnitude of the ES statistics was considered trivial <0.20; small, 0.20–0.50; medium, 0.5–0.80; large, 0.8–1.30; or very large >1.30 [20]. The effect size is reported in conjunction with the 95% confidence interval (CI) for all analyzed measures. The significance level was set at *p* ≤ 0.05.

## 3. Results

The energy, carbohydrate, lipid, and protein intakes did not differ between the before and after intervention measurements for the HMB-FA and placebo groups (Table 1). There were also no significant differences between groups in age, height, body mass and BMI.

**Figure 2:**
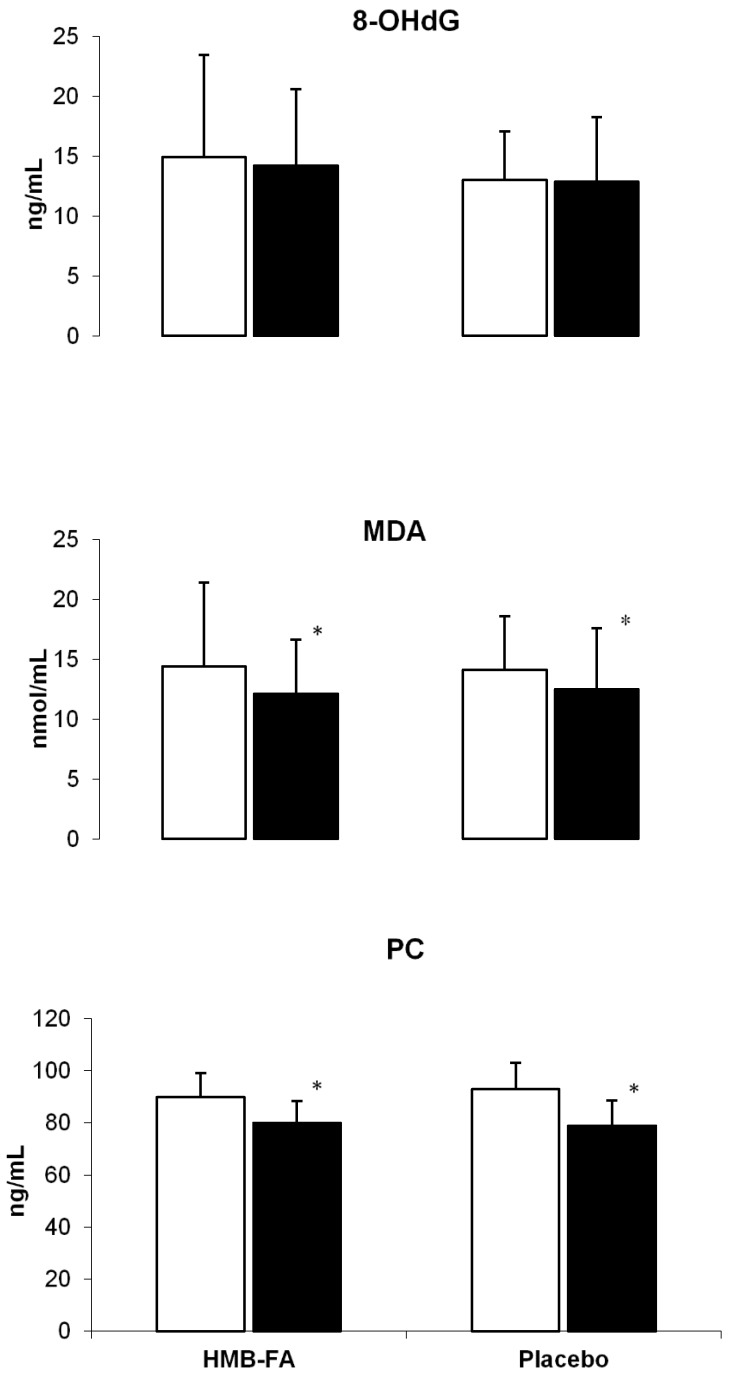
Changes in oxidative stress markers following 6 weeks of a training intervention (means ± SDs).*: denotes significant differences between baseline and post training values (*p* ≤ 0.05); (□ pre-test, ■ post-test); 8-OHdG: 8-hydroxy-2-deoxyguanosine; MDA: malondialdehyde; PC: protein carbonyl.

Changes in the serum 8-OHdG, MDA and PC are presented in Figure 2. No significant time or group by time changes in 8-OHdG were observed after 6 weeks of training. 

The serum MDA decreased significantly (*p* = 0.01) in the HMB-FA supplementation (from 14.4 ± 7 to 12.2 ± 4.5 nmol/mL; ES = 0.39, 95% CI = −0.62 to 1.35) and placebo supplementation (from 14.2 ± 4.5 to 12.5 ± 5.1 nmol/mL; ES = 0.33, 95% CI = −0.66 to 1.31) groups after 6 weeks of training. Both groups showed very large, significant (*p* = 0.05) decrements in the serum PC levels (HMB-FA: from 89.9 ± 9.1 to 77.9 ± 8.4 ng/mL; ES = −1.37, 95% CI = 0.22 to −2.37; placebo: from 92.8 ± 10.2 to 78.8 ± 9.6 ng/mL; ES = −1.41, 95% CI = 0.25 to −2.42) after training. No statistically significant group × time interactions in MDA (*p* = 0.61) or PC (*p* = 0.37) were observed after the training intervention.

The HMB-FA and placebo groups demonstrated large, significant (*p* = 0.05) decreases in ALT after 6 weeks of training. In addition, both groups showed medium decrements (*p* = 0.04) in AST (HMB-FA group, ES = −0.59; placebo group, ES = −0.58) and trivial to small changes (HMB-FA group, ES = −0.02, *p* = 0.1; placebo group, ES = 0.29, *p* = 0.04) in ALP after 6 weeks of training. For WBC, the HMB-FA and placebo groups achieved small to trivial significant decreases. Trivial to medium changes in neutrophils, lymphocytes, and monocytes were observed after resistance training for the HMB-FA and placebo groups (Table 2). No statistically significant group × time interactions in biochemical variables (*p* > 0.05) were observed after the training intervention.

## 4. Discussion

The aim of this study was to investigate the effects of 6 weeks of resistance training, together with HMB-FA supplementation, on oxidative stress and biochemical variables in humans. The results of this study demonstrate that 6 weeks of resistance training can attenuate oxidative stress and biochemical variables after resistance training and HMB-FA supplementation does not induce further adaptive changes in oxidative stress markers.

In the present study, serum 8-OHdG did not change after training for both the HMB-FA and placebo groups. In contrast, MDA and PC levels significantly decreased after 6 weeks of training in both groups. This finding demonstrates that HMB-FA supplementation with resistance training can result in possible decreases in MDA. Very few studies have examined the effects of resistance training on MDA levels [4,5,6] and to the authors knowledge, no studies have investigated the influence of HMB-FA supplementation on MDA changes following long duration training. In accordance with our findings, Cakir-Atabek et al. [21] reported that 6 weeks of resistance training reduced resting MDA levels. In addition, Azizbeigi et al. [4] demonstrated that 8 weeks of progressive resistance training induced significant decreases in resting MDA levels in men. A potential mechanism for the resistance training-induced reduction of MDA could include contraction-induced antioxidant enzyme upregulation [22]. Moreover, the antioxidant effects of exercise may not only be mediated by increased expression of antioxidant enzymes, but may also include reduced expression of pro-oxidant enzymes [23]. Regarding the minimal differences between the HMB-FA and placebo groups in terms of MDA decrement (ES; 0.39, 15.3% vs. 0.33, 12%) after resistance training, it seems that, the Cholesterol Synthesis Hypothesis (CSH) could be proposed as a possible mechanism [23]. However, in this study we did not measure any variables related to the CSH, although Nissen et al. [23] reported that muscle cell damage after resistance exercise may cause cells to lack the capacity to produce adequate amounts of cholesterol to maintain sarcolemma integrity. This is important for muscle tissue. Cholesterol is formed from acetyl-CoA, catalyzed by the enzyme, HMG-CoA reductase. The majority of HMB is converted into HMG-CoA reductase [23]. Therefore, supplementation with HMB-FA may increase the intramuscular HMB concentration and may provide greater available substrate for cholesterol synthesis, which is needed to stabilize the sarcolemma and inhibit cell membrane breakdown and consequently, prohibit MDA production in the blood serum.

The amount of protein carbonyl (PC) was decreased for both the HMB-FA and placebo groups after 6 weeks of resistance training (ES; −1.37 vs. −1.41). This finding is in line with previous studies which have reported decrements in protein carbonylation after long term resistance training [4,7]. The physiological consequences of the carbonylation of proteins are dependent on the kind of protein affected by oxidation [5]. Training-induced protein oxidation might also be triggered by iron-containing protein disruption [24]. A calcium homeostasis imbalance may also cause ROS generation through activation of phospholipase and proteolytic enzymes [24]. However, it is difficult to say which proteins and pathways are associated, since proteins may differ greatly in their susceptibility to oxidative damage and carbonylation of proteins. In this study, we used hypertrophic-type resistance training, and this type of training could improve antioxidant levels in the body [4,7] resulting in a decrement of PC. Regarding the lower changes in PC levels for the HMB-FA compared with the placebo group, it seems that HMB supplementation may inhibit intracellular protein degradation and decrease the ubiquitin–proteasome proteolysis pathway [23,25], resulting in a restriction of the carbonylation of proteins. 

For the biochemical variables, the HMB-FA and placebo groups exhibited similar decrements in ALT and AST (large to medium ES), while the placebo group showed a greater increase in ALP after 6 weeks of resistance training (ES; 0.29 vs. −0.02). Although both groups showed significant decreases in WBC, the HMB-FA group indicated greater training effects than the placebo group (ES; −0.49 vs. −0.19). The 6 weeks of resistance training did not induce any significant effects in neutrophils or lymphocytes for the HMB-FA group, while the placebo group showed significant increases in neutrophils after 6 weeks. In monocytes, both groups showed slight decrements after 6 weeks of training. A possible potential mechanism(s) for the resistance training-induced reduction of biochemical variables could be due to upregulation of antioxidant enzymes following chronic resistance training and decrements in the expression of pro-oxidant enzymes [4,7].

These findings reveal that supplementation with HMB does not induce enough protective effects for biochemical variables and further studies are needed to determine the long-term effects of HMB-FA supplementation on inflammation after resistance training. 

## 5. Conclusions

In conclusion, the results of this study demonstrated that 6 weeks of resistance training could decrease oxidative stress markers in men. Separately, HMB-FA supplementation has a similar effect on the inhibition of oxidative stress markers and biochemical variables; however, regarding ES, the HMB-FA group exhibited greater training effects than the placebo group. Resistance training can be considered an effective therapeutic intervention to reduce oxidative stress, and HMB-FA supplementation during resistance training does not add further adaptive changes related to oxidative stress markers.

## Figures and Tables

**Table 1:** Dietary intake assessed for the beta-hydroxy-beta methylbutyrate-free acid (HMB-FA) and placebo supplementation groups before and after the training period. Data are presented as means ± SDs.

Diet Variables	Time	HMB-FA (*n* = 8)	Placebo (*n* = 8)
Energy intake (kcal)	Before	2532 ± 210	2421 ± 176
	After	2891 ± 198	2782 ± 215
Carbohydrate (g)	Before	260 ± 23	259 ± 29
	After	282 ± 31	278 ± 41
Fat (g)	Before	75 ± 11	79 ± 13
	After	84 ± 14	81 ± 17
Protein (g)	Before	98 ± 19	93 ± 21
	After	119 ± 22	115 ± 34
Vitamin E (mg)	Before	8.9 ± 1.0	9.0 ± 2.0
	After	10.0 ± 2.0	9.5 ± 1.6
Vitamin C (mg)	Before	65 ± 22	67 ± 17
	After	77 ± 18	78 ± 15

**Table 2:** Changes in biochemical variables following 6 weeks of a training intervention.

Blood Variables		HMB-FA Group		Placebo Group	
		Mean ± SD		Mean ± SD	
ALT (U/L)	Pre	31.1 ± 14.2		34.5 ± 19.8	
	Post	20.1 ± 8.2	*	20.4 ± 7.3	*
	Effect size	−0.94 (−1.92, 0.14)	Large	−0.95 (−1.92, 0.13)	Large
AST (U/L)	Pre	30.2 ± 13.2		30.8 ± 12.2	
	Post	23.7 ± 8.5	*	24.7 ± 8.4	*
	Effect size	−0.59 (−1.56, 0.44)	Medium	−0.58 (−1.55, 0.45)	Medium
ALP (U/L)	Pre	166.0 ± 29.3		164.2 ± 36	
	Post	165.3 ± 49.0		174.6 ± 36.1	*
	Effect size	−0.02 (−1.00, 0.96)	Trivial	0.29 (−0.71, 1.26)	Small
WBC (µ/L)	Pre	8312.5 ± 1817.7		8400.0 ± 900.8	
	Post	7600.0 ± 1033.7	*	8062.5 ± 1924.2	*
	Effect size	−0.48 (−1.45, 0.54)	Small	−0.19 (−1.20, 0.77)	Trivial
Neutrophils (%)	Pre	51.4 ± 10.3		49.5 ± 8.6	
	Post	52.3 ± 9.5		54.5 ± 9.0	*
	Effect size	0.09 (−0.89, 1.07)	Trivial	0.57 (−0.46, 1.53)	Medium
Lymphocytes (%)	Pre	35.8 ± 9.4		35.7 ± 6.5	
	Post	35.0 ± 8.4		34.5 ± 5.7	
	Effect size	0.09 (−0.89, 1.07)	Trivial	−0.02 (−1.17, 0.8)	Trivial
Monocytes (%)	Pre	9.0 ± 1.5		8.9 ± 1.2	
	Post	8.2 ± 1.1	*	7.9 ± 1.6	*
	Effect size	−0.61 (−1.57, 0.43)	Medium	−0.71 (−1.68, 0.34)	Medium

Notes: ALT = alanine transaminase; AST = aspartate aminotransferase; ALP = alkaline phosphatase; WBC = white blood cells; * = significant difference compared to pre-training (*p* ≤ 0.05); effect sizes are presented with 95% confidence intervals.
